# Serum neutrophil gelatinase-associated lipocalin the estimation of hospital prognosis in patients with ST-elevated myocardial infarction

**DOI:** 10.1371/journal.pone.0180816

**Published:** 2017-07-24

**Authors:** Victoria Karetnikova, Anastasia Osokina, Olga Gruzdeva, Evgenya Uchasova, Michael Zykov, Victoria Kalaeva, Vasiliy Kashtalap, Kristina Shafranskaya, Olga Barbarash

**Affiliations:** 1 Federal State Budgetary Institution “Research Institute for Complex Issues of Cardiovascular Diseases”, Kemerovo, the Russian Federation; 2 Federal State Budget Educational Institution of Higher Professional Education “Kemerovo State Medical Academy” the Ministry of Health of the Russian Federation, Kemerovo, the Russian Federation; Osaka University Graduate School of Medicine, JAPAN

## Abstract

We aimed to assess the clinical significance of serum levels of neutrophil gelatinase-associated lipocalin (sNGAL) for predicting in-hospital outcomes in patients with ST-elevated myocardial infarction (STEMI). Patients admitted within 24 hours of developing STEMI clinical symptoms were evaluated for sNGAL on hospitalization days 1 and 12. Recurrent myocardial infarction, early post-infarction angina, acute cerebrovascular accident, and death were assessed as adverse outcomes during hospitalization. The actors associated with adverse outcome were evaluated using univariate and multivariate regression analysis. Among the 260 STEMI patients included, 32% had ≥1 adverse in-hospital outcome, and significantly higher sNGAL on day 12, (but not on day 1) compared to sNGAL in patients with favorable outcome (*p* = 0.033). Type-2 diabetes mellitus, age > 60 years, reduced glomerular filtration rate during hospitalization, and high sNGAL on day 12 were identified as risk factors for adverse in-hospital outcome, associated with a 14% increase for each 1-year increment in age after 60 years, and a dramatic increase (3.2 times) for high sNGAL on day 12, with sNGAL ≥ 1.046 ng/ml indicating complicated hospitalization course. sNGAL concentration on the 12th day was associated with the existing adverse outcomes, acting as a marker of MI severity.

## Introduction

Assessing the risk of adverse outcomes in patients with ST-elevated myocardial infarction (STEMI) represents a tasks in modern cardiology, and requires to analyze not only anamnestic data and the characteristics of the index coronary event, but also the records of ongoing or progressing organ failures, which may complicate the course of the disease [1].

STEMI patients are at a particularly high risk of acute kidney injury (AKI) because of the complexity of hemodynamic disorders and adverse effects associated with the use of radiopaque diagnostic and treatment methods [1]. AKI is typically diagnosed based on the assessment of glomerular filtration rate (GFR) and creatinine clearance rate. Nevertheless, in the context of current diagnostic capabilities, biomarkers represent a promising alternative for the diagnosis of myocardial infarction and prediction of its outcomes. Using biomarkers is advantageous because these indicators are universal and allow to estimate early prognosis, as it is possible to assess not only the presence of kidney injury or the severity of comorbid pathologies, but also the risk of cardiovascular events.

Neutrophil gelatinase-associated lipocalin (NGAL) can serve as such a marker, as it is considered one of the most accurate markers acute kidney injury [2]. However, only limited data are available regarding its value in predicting outcomes in STEMI patients. Therefore, the purpose of the present study was to assess the clinical significance of NGAL as a tool for predicting in-hospital outcomes for patients with STEMI.

## Materials and methods

### Study protocol

This was a retrospective evaluation of patients admitted between 2008 and 2010 to the Kemerovo Cardiology Hospital, in Kemerovo, Russia, with acute coronary syndrome characterized by ST-segment elevation. Data were extracted from the Russian registry of acute coronary syndrome (RECORD) [3].

The study protocol, which received approval from the Ethics Committee of the Research Institute for Complex Issues of Cardiovascular Diseases, was developed in accordance with the Declaration of Helsinki of the World Medical Association (“Ethical Principles for Medical Research Involving Human Subjects”), ammended in 2000, as well as with the “Rules of clinical practice in the Russian Federation”, approved by Order of the Ministry of Health of the Russian Federation (266/19.06.2003). Patient enrollment in the study was voluntary, and each patient provided written informed consent for participation.

### Patients

Patients were also included if they had at least two of the following criteria (including elevated biochemical markers of myocardial necrosis): (1) clinical findings of chest pain lasting >20 min; (2) electrocardiographic (ECG) findings with ST-segment elevation on ECG in two or more contiguous leads with the cut-off point of ≥0.1 mV or complete left bundle branch block; and (3) biochemical findings of elevated troponin T levels ≥0.1 ng/ml and/or creatine kinase-MB isoenzyme levels ≥25 IU/l.

The exclusion criteria were: age under 18 years; myocardial infarction requiring complicated percutaneous coronary intervention (PCI) or coronary artery bypass grafting (CABG); mental illnesses; comorbidities significantly affecting the outcome and prognosis, including cancer, terminal hepatocellular insufficiency, acute infectious diseases, or advanced chronic diseases.

### Data collection

The following evaluations were performed for all patients: medical history; physical examination; 16-lead electrocardiography; echocardiography with the assessment of left ventricular ejection fraction (LVEF) and zones of regional contractility violation; blood tests for estimating the levels of troponin T, total creatine phosphokinase and its MB-isohorm, hemoglobin, creatinine, glucose, total cholesterol and lipid spectrum, and serum NGAL (sNGAL). Blood sampling for estimation of sNGAL concentration was collected after radiopaque intervention.

Evaluation of sNGAL (ng/ml) on the 1^st^ and 12^th^ day of hospitalization was performed via an enzyme-linked immunosorbent assay (Hycult Biotech, Uden, The Netherlands), and the results were recorded using a flatbed reader (UNIPLAN; SPC PIKON, Russia). The reference range for sNGAL values was considered at under 0.4 ng/ml.

GFR was estimated using the formula established via the Modification of Diet in Renal Disease study, [4] taking into account the serum creatinine levels, and was recorded from admission until discharge, inclusively (10–14 days). Cases with GFR decline to less than 60 ml/min/1.73 m^2^ were noted.

The diagnosis of diabetes mellitus (DM) and/or chronic kidney disease (CKD) was extracted from the patients’ medical history records and out-patient medical records, but were also confirmed based on carbohydrate metabolism indicators and creatinine-based GFR recorded during index hospitalization.

For all patients, the modality of reperfusion therapy was established upon admission as follows: PCI in the form of angioplasty and/or stenting of the culprit coronary artery; thrombolytic therapy; or no reperfusion therapy, if common contraindications or technical constraints were present. The time from the onset of chest pain to balloon PCI was 78–11.2 mins.

Totally 109 endpoints were identified: recurrent myocardial infarction– 38 cases, early post-infarction angina– 42, stroke– 4, progressive acute heart failure (increase in Killip class at least by one) was noted in 11cases, mortality– 14; combined endpoints (more than 1 event)– 16.

The following clinical indicators were assessed during hospitalization: manifestations of coronary insufficiency, including development of early post-infarction angina or recurrent myocardial infarction; degree of acute heart failure (AHF), expressed in terms of Killip grade (I–IV); chronic heart failure (CHF) class, according to the New York Heart Association Functional Classification; and in-hospital mortality.

### Statistical analyses

Statistical processing of the data was performed using SPSS version 16.0 (SPSS Inc., Chicago, IL, USA). The Pearson criterion (χ^2^) was used to analyze the differences in frequency. The calculation of odds ratio (OR) with 95% confidence interval (95% CI) was performed using the corresponding option in the program. Two independent groups were compared using the Mann–Whitney U-test. The Kruskal-Wallis test was used to compare several independent groups. To identify the independent predictors of adverse outcomes, the logistic regression method was applied. The differences were considered statistically significant for *p*-values less than 0.05.

## Results

The study included 260 patients (mean age at admission, 57 years; age range, 51–64 years) admitted between January 2008 and December 2010. The baseline characteristics of all patients included in the study are provided in Table 1.

**Table 1:** Baseline characteristics of male patients admitted with ST-elevated myocardial infarction (n = 260).

Indicator	Value
Age, years	57 (51; 64)
>60 years old	96 (37)
BMI, kg/m^2^	27.4 (24.7; 30.2)
BMI >25 kg/m^2^	186 (71.5)
DM type 2	23 (8.8)
Arterial hypertension	210 (80.7)
Hypercholesterolemia	55 (21.1)
Smoking	164 (63)
CVA history	19 (7.3)
CAD family history	63 (24.2)
Angina pectoris history	107 (41.1)
PICS	45 (17.3)
CHF history	38 (14.6)
LVEF, %	50 (45; 56)
LVEF < 40%	38 (14.6)
Kidney disease history	114 (43.8)
Gout	27 (10.4)
MI of anterior localization	123 (47.3)
Killip I	225 (86.5)
Killip II-IV	35 (13.5)
PCI	202 (77.7)
Regular medication prescription before admission	
Aspirin	111 (42.7)
Clopidogrel	17 (6.5)
β-blockers	105 (40.4)
ACE inhibitors	90 (34.6)
Angiotensin II receptor blockers	9 (3.4)
Statins	98 (37.7)
Nitrates	18 (6.9)
Diuretics	17 (6.5)
Aldosterone receptor antagonists	28 (10.7)
Calcium channel blockers	62 (23.8)
Antiarrhythmics	16 (6.1)

Data given as mean (25th; 75th percentile) or total number (percentage).

BMI: body mass index; DM: diabetes mellitus; PICS: post-infarction cardiosclerosis; CHF: chronic heart failure; CVA: acute cerebrovascular accident; CAD: coronary artery disease; LVEF: left ventricular ejection fraction; PCI: percutaneous coronary intervention; ACE: angiotensin-converting-enzyme; MI: myocardial infarction.

All patients were admitted within 24 hours of the moment of manifestation of clinical symptoms of STEMI. The study included only male patients so as to exclude the potential sex-specific impact on our assessment of STEMI outcomes. DM diagnosis was verified in 23 patients (8.8%), based on medical history data and laboratory parameters (fasting and postprandial glycaemia). The following notable cardiovascular risk factors were found: arterial hypertension (80.7%), overweight status (71.5%), and smoking status (63.0%). Aspirin, β blockers, angiotensin-converting-enzyme inhibitors, and statins were among the most common drugs taken routinely before admission. Myocardial infarction of anterior localization was diagnosed in 47.3% of cases, and the signs of acute heart failure indicated Killip grade I in most patients (86.5%).

The patients were stratified into two groups depending on the occurrence of adverse in-hospital outcomes including recurrent myocardial infarction, early post-infarction angina, acute cerebrovascular accident (CVA), and death. Adverse in-hospital outcome (i.e., the presence of at least one of the above-described outcomes) was noted in 83 patients (32%), who were included in the favorable outcome group. The remaining 177 patients (68%) did not experience in-hospital complications, and were included in the favorable outcome group (Table 2).

**Table 2:** Clinical and anamnestic characteristics of male patients admitted with ST-elevation myocardial infarction (MI), stratified according to in-hospital outcomes.

Indicator	Favorable in-hospital outcome(n = 177)	Unfavorable in-hospital outcome(n = 83)	*p*-value
Age, years	57 (50; 62)	60 (53; 68)	0.002
>60 years old	57 (32.2)	39 (47)	0.021
BMI, kg/m^2^	27.4 (25; 30.1)	27 (23.5; 30.7)	0.62
BMI > 25 kg/m^2^	134 (75.5)	52 (62.2)	0.073
DM type 2	10 (5.6)	13 (15.6)	0.008
Arterial hypertension	138 (77.9)	72 (86.7)	0.09
Hypercholesterolemia	36 (20.3)	19 (22.9)	0.53
Smoking	112 (63.3)	52 (62.2)	0.92
CVA history	9 (5)	10 (12)	0.044
CAD family history	49 (27.7)	14 (16.8)	0.03
Angina pectoris history	65 (36.7)	42 (50.6)	0.034
PICS	30 (17)	15 (18)	0.82
CHF history	22 (12.4)	16 (19.3)	0.14
LVEF, %	50 (45; 57)	50 (42; 55)	0.16
LVEF < 40%	66 (37.3)	17 (20.5)	0.066
CKD history	68 (38.4)	46 (55.4)	0.010
Gout	18 (10.2)	9 (10.8)	0.90
MI characteristics on admission			
MI of anterior localization	80 (45.2)	43 (51.8)	0.31
Killip I	169 (95.5)	56 (67.4)	0.0000
Killip II-IV	8 (4.5)169	27 (32.5)56	0.0000
PCI	143 (80.8)	59 (71.1)	0.079
CAG	174 (98.3)	82 (98.8)	0.76
In-hospital medication prescription			
Aspirin	82 (46.3)	29 (34.9)	0.93
Clopidogrel	11 (6.2)	6 (7.2)	0.31
β-blockers	80 (45.2)	25 (30.1)	0.31
ACE inhibitors	66 (37.3)	24 (28.9)	0.59
Angiotensin II receptor blockers	6 (3.4)	3 (3.6)	0.57
Statins	77 (43.5)	21 (25.3)	0.041
Nitrates	12 (6.8)	6 (7.2)	0.41
Diuretics	9 (5)	8 (9.6)	0.027
Aldosterone receptor antagonists	22 (12.4)	6 (7.2)	0.58
Calcium channel blockers	45 (25.4)	17 (20.5)	0.64
Antiarrhythmics	11 (6.2)	5 (6.02)	0.56

Data given as mean (25th; 75th percentile) or total number (percentage).

BMI: body mass index; DM: diabetes mellitus; PICS: post-infarction cardiosclerosis; CHF: chronic heart failure; CVA: acute cerebrovascular accident; CAD: coronary artery disease; CAG: coronary angiography; ACE: angiotensin-converting-enzyme; LVEF: left ventricular ejection fraction; PCI: percutaneous coronary intervention.

In our study, 14 patients (5.1%) died during hospitalization (up to 12 days). The in-patient treatment stage of up to 12 days was completed by 88 patients, but blood sampling was performed in 84 patients at the indicated time, as well as in 172 patients who were hospitalized during this period. Thus, in total, 256 blood samples (93.4%) were analyzed on the 12^th^ day of myocardial infarction. These aspects were clarified in the revised manuscript.

Comparison between the groups indicated that patients older than 60 years prevailed in the group with adverse outcome (47% vs 32.2%, *p* = 0.002). In the same group, the index myocardial infarction was significantly more likely to be associated with a history of CVA (12% vs 5%, *p* = 0.044), clinical findings of angina (50.6% vs 36.7%, *р* = 0.034), and kidney disease diagnosed at the out-patient treatment stage (55.4% vs 38.4%, *р* = 0.01).

The unfavorable outcome group contained 2.8 times more patients with type 2 DM than did the favorable outcome group (15.6% vs 5.6%; *p* = 0.008). AHF of Killip grade II-IV class was diagnosed 7 times more often in the unfavorable outcome group (32.5% vs 4.5%; *p* < 0.05).

In terms of the medication use during hospitalization, we noted more frequent use of diuretics (9.6% vs 5%; *p* = 0.027), as well as less frequent prescription of statins (43.5% vs 25.3%; *p* = 0.041) in the unfavorable outcome group; the groups did not differ regarding the in-hospital prescription of other types of medication. The differences in the use of diuretics may be explained by the fact that a greater number of patients with Killip grade II-IV were included in the unfavorable outcome group.

The two groups also differed in terms of clinical and laboratory parameters (Table 3).

**Table 3:** Laboratory findings in patients admitted with ST-elevated myocardial infarction (n = 260).

Indicators	Favorable in-hospital outcome(n = 177)	Unfavorable in-hospital outcome(n = 83)	*p*-value
Cholesterol, mmol/l	5.2 (4.3; 5.8)	5 (4.3; 5.7)	0.67
HDL, mmol/l	1.4 (0.9; 1.2)	1.1 (0.9; 1.2)	0.18
LDL, mmol/l	2.9 (2.2; 3.8)	2.7 (2.3; 3.3)	0.29
TG, mmol/l	1.8 (1.4; 2.4)	1.6 (1.2; 2.4)	0.30
AI	3.8 (2.9; 4.7)	3.4 (2.7; 4.5)	0.19
Hemoglobin, g/l	142 (134; 151)	144 (137; 152)	0.38
Anaemia	25 (14.1)	9 (10.8)	0.46
Proteinuria	27 (15.2)	13 (15.6)	0.82
Glucose, mmol/l	6.2 (5.8; 6.7)	7.8 (7.1; 10.6)	0.049
Blood creatinine before CAG, umol/l	97 (86; 108)	97 (87; 107)	0.82
Blood creatinine after CAG, umol/l	91.5 (81; 112)	120 (115; 128)	0.051
Blood creatinine before discharge, umol/l	93 (83; 106)	99 (90; 110)	0.023
GFR by MDRD, ml/min/1.73m^2^	74.2 (63.9; 86.6)	73.7 (62.1; 86.1)	0.87
GFR increase during hospitalization	22 (12.4)	11 (13.2)	0.15
GFR decrease during hospitalization	8 (4.5)	15 (8)	0.0003
GFR < 60 ml/min/1.73m^2^ before discharge	27 (12.4)	11 (13.2)	0.15
sNGAL on the 1^st^ day, ng/ml	1.36 (0.26; 2.27)	1.53 (1.19; 2.26)	0.32
sNGAL on the 12^th^ day, ng/ml	1.55 (1.11; 2.3)	2.1 (1.44; 2.8)	0.033

Data given as mean (25th; 75th percentile) or total number (percentage).

HDL: high-density lipoproteins; LDL: low-density lipoproteins; TG: triglycerides; AI: atherogenic index; CAG: coronary angiography; GFR: glomerular filtration rate; MDRD: Modification of Diet in Renal Disease study; sNGAL: serum levels of neutrophil gelatinase-associated lipocalin.

Specifically, blood glucose levels on admission appeared to be higher in the unfavorable outcome group (7.8 vs 6.2 mmol/l; *p* = 0.04). The levels of blood creatinine over the course of hospitalization and at discharge were higher in the unfavorable outcome group (116 vs 105.5 umol/l, with *p* = 0.009; 99 vs 93 umol/l, with *p* = 0.023; respectively). A decrease in GFR to under 60 ml/min/1.73m^2^ during hospitalization was observed 2 times more often in the unfavorable outcome group (8% vs 4.2%; *p* = 0.0003).

**Fig 1:**
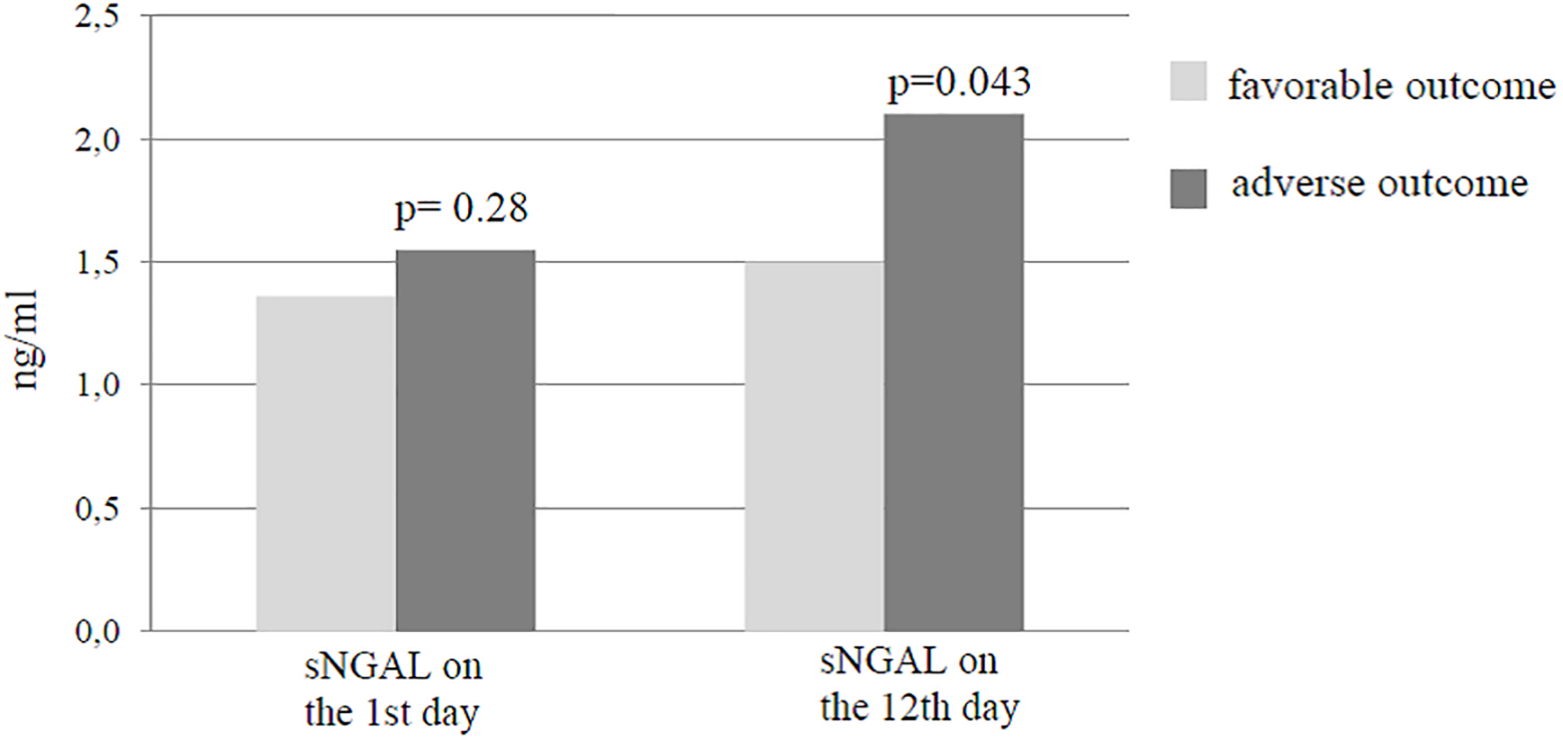
Relationship between in-hospital outcomes and serum levels of neutrophil gelatinase-associated lipocalin (sNGAL) in male patients admitted with ST-elevated myocardial infarction. p—value for differences between groups (p < 0.05).

Interestingly, the levels of the kidney injury biomarker sNGAL noted for hospitalization day 1 did not differ between the groups. Nevertheless, sNGAL for hospitalization day 12 appeared to be significantly higher in the unfavorable outcome group (2.1 vs 1.55 ng/ml; *p* = 0.033; Fig 1).

The correlation analysis suggestedhowed the presence of a direct positive association between sNGAL on hospitalization day 12 and the presence of CKD history, early post-infarction angina, left ventricular systolic dysfunction (LVEF < 40%; Table 4).

**Table 4:** Correlation between clinical-anamnestic parameters and serum levels of neutrophil gelatinase-associated lipocalin (sNGAL) estimated on the 12^th^ day of hospitalization for male patients admitted with ST-elevated myocardial infarction (STEMI).

Parameter	sNGAL on the 12^th^ day
Kidney disease history	*r* = 0.33, *р* = 0.009
LVEF < 40%	*r* = 0.26, *р* = 0.04
Early post-infarction angina	*r* = 0.32, *р* = 0.012
Fasting glycaemia on the 2^nd^ and the 3^rd^ day of STEMI	*r* = 0.33, *р* = 0.051

LVEF: left ventricular ejection fraction

Univariate analysis showed that the presence of type 2 DM, age > 60 years, significant GFR decrease during hospitalization, and higher sNGAL on hospitalization day 12 were significant risk factors for the development of adverse outcomes hospitalization (Table 5).

**Table 5:** Logistic regression analysis of the factors contributing to adverse hospital outcome in male patients admitted with ST-elevated myocardial infarction.

Indicator	OR	95% CI	*p*-value
Univariate analysis			
Age > 60 years old	1.86	1.09–3.19	0.022
GFR decrease during hospitalization	1.7	1.0–2.9	0.035
DM type 2	1.78	1.0–3.19	0.049
sNGAL on the 12^th^ day	9.8	1.2–119	0.047
Multivariate analysis			
Age > 60 years old	1.14	1.10–1.19	0.037
sNGAL on the 12^th^ day	3.2	1.4–7.8	0.048

DM: diabetes mellitus; GFR: glomerular filtration rate; sNGAL: serum levels of neutrophil gelatinase-associated lipocalin; OR: odds ratio; 95% CI: 95% confidence interval

Specifically, GFR decrease during hospitalization, presence of type 2 DM, and significantly higher sNGAL on hospitalization day 12 were associated with a 1.9-, 1.8-, and 10-fold increase in the incidence of adverse outcomes.

By the method of multivariate logistic regression was applied on the set of predictors identified via univariate analysis to have a significant impact on in-hospital outcomes. The predictors were included into the final model in a stepwise manner, and the following effects were noted: an 1-year increment in age in patients over 60 years old increases the risk of adverse outcomes by 14%; higher sNGAL on hospitalization day 12 is associated with a 3.2-fold increase in risk for adverse outcome (Table 5).

The receiver operating characteristic analysis of sNGAL impact on the risk of adverse hospital outcomes revealed an area under the curve of 0.703 (95% CI: 0.43–0.97), suggesting that our risk prediction model has high quality. The threshold for significant risk was found at sNGAL ≥ 1.046 ng/ml, which was associated with a complicated hospitalization course of myocardial infarction.

## Discussion

The value of sNGAL as a marker of acute kidney injury is well established, [5] as is its role in the assessment of the severity of kidney injury. Thus, according to some authors, increased levels of this marker can be regarded as one of the indications for hemodialysis [6]. Moreover, the independent association of sNGAL with renal failure and progression of CKD was also noted, which likely underlies the high prognostic value of this marker [7]. The relationship between CKD and cardiovascular diseases is known; specifically, the maximum risk of developing cardiovascular disease, as well as the risk of experiencing an unfavorable course of the ongoing cardiovascular disease have been determined among patients with end-stage renal disease [8]. Furthermore, there is evidence regarding the association between increased sNGAL and total mortality rates in elderly individuals [9].

NGAL is secreted by the epithelium of renal tubules in response to kidney injury, and this process precedes the increase in serum creatinine concentration [8]. On the basis of this feature, NGAL is regarded as a specific and sensitive marker of AKI, in particular after cardiosurgical interventions and administration of radiopaque agents [10]. The prognostic value of sNGAL, previously demonstrated as an accurate marker of kidney damage in terms of unfavorable 30-day prognosis in AHF patients, significantly exceeds that of creatinine; thus, sNGAL may be used as an indicator to complement assessments of the levels of brain natriuretic peptide [11]. The predictive role of NGAL was also shown in patients admitted to the emergency department with AKI, but who did not have any history of kidney disease [12]. However, the role of sNGAL in the prediction of STEMI prognosis remains under discussion. In our present study, sNGAL on hospitalization day 12, but not that on day 1, correlated with in-hospital outcomes; specifically, higher sNGAL on hospitalization day 12 was associated with dramatically increased occurrence of at least one adverse outcome during hospitalization.

Moreover, the unfavorable outcome group contained a greater proportion of patients with significant reduction in GFR over the course of hospitalization, which is consistent with the findings of Bolignano et al. [13]. and suggests that sNGAL may serve as an indicator of residual renal function. According to the “forest fire” theory, the relationship between higher sNGAL and decrease in GFR is related to the fact that NGAL production takes place in viable cells of the inflammatory-modified tubular epithelium, whereas the increase in creatinine concentration and decrease in GFR reflect only the total, irreversible loss of functional nephrons [14]. Viewed from this perspective, sNGAL can be considered to characterize the residual volume of functional nephrons.

In addition, the correlations noted between sNGAL on hospitalization day 12 and other indicators of severe cardiovascular dysfunction including LVEF < 40% and presence of early post-infarction angina, as well as the absence of similar associations concerning sNGAL on hospitalization day 1, highlights the role of sNGAL as a marker of unfavorable course during hospitalization for STEMI. The complications noted during the hospitalization period may represent an independent cause of AKI, and may lead to additional secretion of NGAL.

## Conclusions

Using the one-dimensional and multidimensional models, we also demonstrated the value of sNGAL as a factor reflecting the unfavorable course of myocardial infarction. The identified trends suggest that sNGAL can serve as a marker of infarction severity and unfavorable course in the early period of hospitalization for STEMI, and that an increase in sNGAL should be considered a serious development even in patients with absent or minimal clinical manifestations.

### Ethical approval

The study protocol was approved by the local ethics committee of the Federal State Budgetary Scientific Institution Research Institute for Complex Issues of Cardiovascular Diseases.

### Limitations of this study

This subgroup analysis has limitation. It should be mentioned that no data concerning the number of peripheral blood leukocytes was available.

## Abbreviations

MI: myocardial infarction
NGAL: neutrophil gelatinase-associated lipocalin
AKI: acute kidney injury
GFR: glomerular filtration rate
PCI: percutaneous coronary intervention
CABG: coronary artery bypass grafting
CVA: cerebrovascular accident
ACS: acute coronary syndrome
DM: diabetes mellitus
CHF: chronic heart failure
AHF: acute heart failure
CKD: chronic kidney disease

## References

[1] Smirnov AV, Kayukov IG, Dobronravov VA, Kucher AG. Acute kidney injury—a new concept in nephrology. Clinical Nephrology. 2009; 1: 11–5. (Russian)

[2] Mukhin NA, Svistunov AA, Fomin VV. Clinical diagnosis progress and continuing medical education. Ther Arkh. 2014; 4: 4–7. (Russian) 24864460

[3] Ehrlich AD. The registers of acute coronary syndromes—their types, characteristics and significance in clinical practice. Vestn Ross Akad Med Nauk. 2012; 4: 30–39. (Russian) 22834325

[4] Levey AS, Stevens AL, Schmid CH, Zhang YL, Castro AF 3rd, Feldman HI, et al A new equation to estimate glomerular filtration rate. Ann Intern Med. 2009; 150: 604–12. 19414839 10.7326/0003-4819-150-9-200905050-00006 PMC2763564

[5] Siew D, Ikizler TA, Gebretsadik T, Shintani A, Wickersham N, Bossert F, Peterson JF, et al Elevated urinary IL-18 levels at the time of ICU admission predict adverse clinical outcomes. Clin J Am Soc Nephrol. 2010; 5: 1497–1505. doi: 10.2215/CJN.09061209 20558561 10.2215/CJN.09061209 PMC2924408

[6] Siew ED, Ware LB, Gebretsadik T, Shintani A, Moons KG, Wickersham N, et al Urine neutrophil gelatinase-associated lipocalin moderately predicts acute kidney injury in critically ill adults. J Am Soc Nephrol. 2009; 20: 1823–1832. doi: 10.1681/ASN.2008070673 19628673 10.1681/ASN.2008070673 PMC2723988

[7] Bolignano D, Lacquaniti A, Coppolino G, Donato V, Campo S, Fazio MR, et al Neutrophil gelatinase-associated lipocalin (NGAL) and progression of chronic kidney disease. Clin J Am Soc Nephrol. 2009; 4: 337–44. doi: 10.2215/CJN.03530708 19176795 10.2215/CJN.03530708 PMC2637601

[8] Zoccali C. The burden of cardiovascular disease in patients with chronic kidney disease and in end-stage renal disease. Contrib Nephrol. 2008; 161: 63–7. doi: 10.1159/000129755 18451659 10.1159/000129755

[9] Daniels LB, Barrett-Connor E, Clopton P, Laughlin GA, Ix JH, Maisel AS. Plasma neutrophil gelatinase-associated lipocalin is independently associated with cardiovascular disease and mortality in community-dwelling older adults: The Rancho Bernardo Study. J Am Coll Cardiol. 2012; 59: 1101–1109. doi: 10.1016/j.jacc.2011.11.046 22421304 10.1016/j.jacc.2011.11.046 PMC3312791

[10] Ling W, Zhaohui N, Ben H, Leyi G, Jianping L, Huili D, et al Urinary IL-18 and NGAL as early predictive biomarkers in contrast-induced nephropathy after coronary angiography. Nephron Clin Pract. 2008; 108: 176–181. doi: 10.1159/000117814 18287807 10.1159/000117814

[11] Maisel AS, Mueller C, Fitzgerald R, Brikhan R, Hiestand BC, Iqbal N, Prognostic utility of plasma neutrophil gelatinase-associated lipocalin in patients with acute heart failure: the NGAL Evaluation Along with B-type NaTriuretic Peptide in acutely decompensated heart failure (GALLANT) trial. Eur J Heart Fail. 2011; 13: 846–51. doi: 10.1093/eurjhf/hfr087 21791540 10.1093/eurjhf/hfr087 PMC3143832

[12] Soto K, Papoila AL, Coelho S, Bennett M, Ma Q, Rodrigues B, et al Plasma NGAL for the diagnosis of AKI in patients admitted from the emergency department setting. Clin J Am Soc Nephrol 2013; 8: 2053–63. doi: 10.2215/CJN.12181212 24009223 10.2215/CJN.12181212 PMC3848412

[13] Bolignano D, Lacquaniti A, Coppolino G, Campo S, Arena A, Buemi M. Neutrophil gelatinase-associated lipocalin reflects the severity of renal impairment in subjects affected by chronic kidney disease. Kidney Blood Press Res/ 2008; 31: 255–58. doi: 10.1159/000143726 18600028 10.1159/000143726

[14] Mori K, Nakao K. Neutrophil gelatinase-associated lipocalin as the real-time indicator of active kidney damage. Kidney Int.2007; 71: 967–70. doi: 10.1038/sj.ki.5002165 17342180 10.1038/sj.ki.5002165

